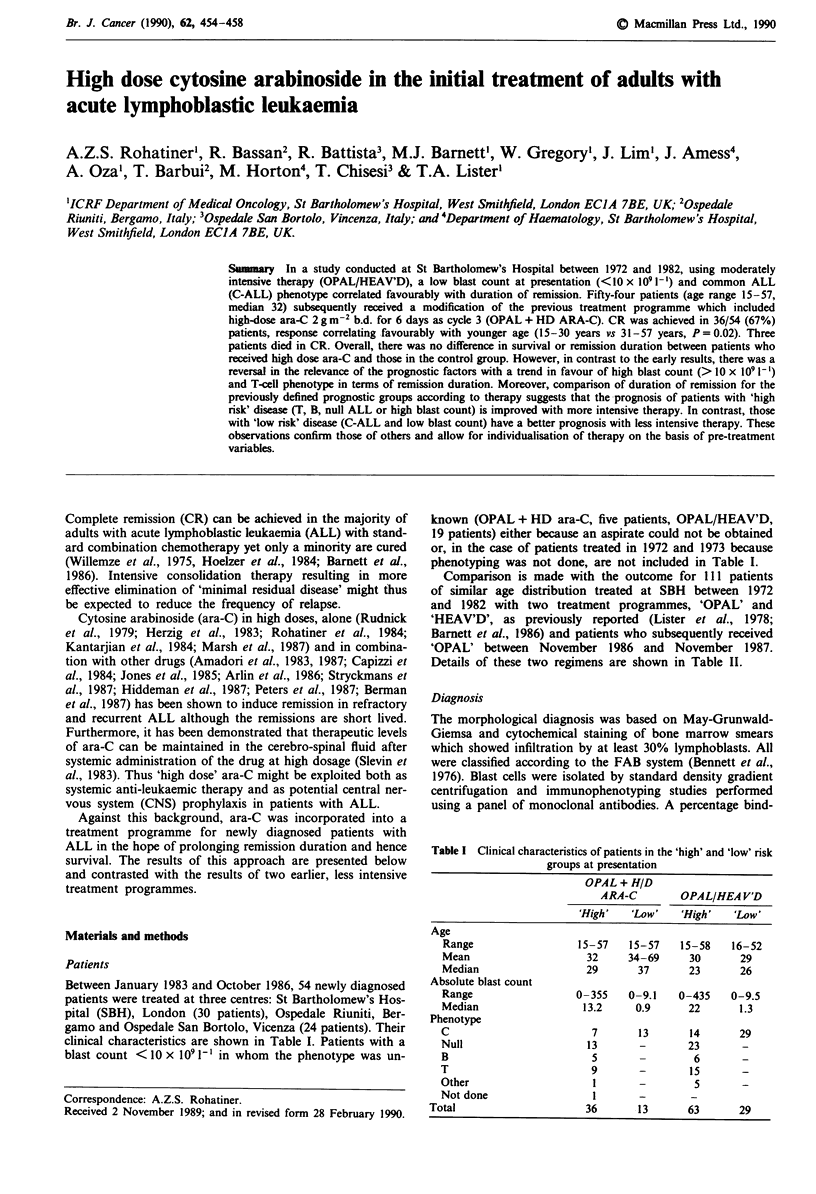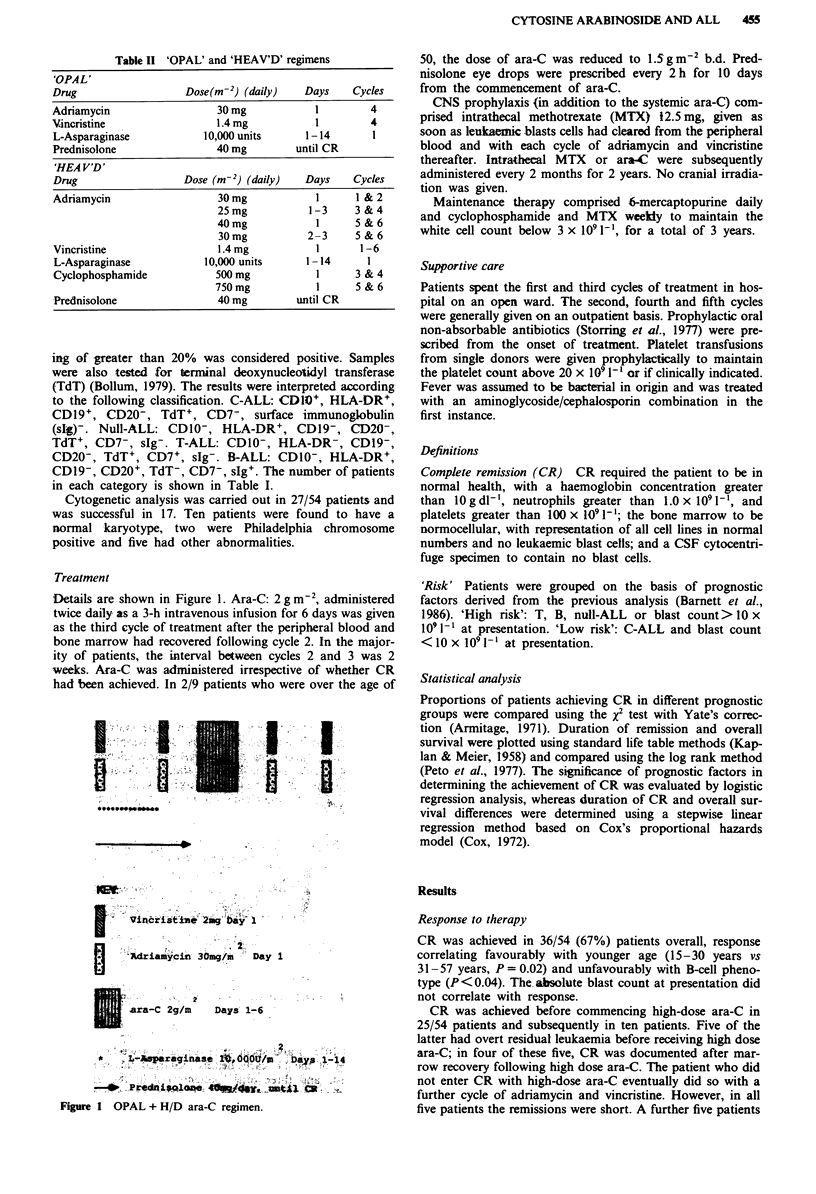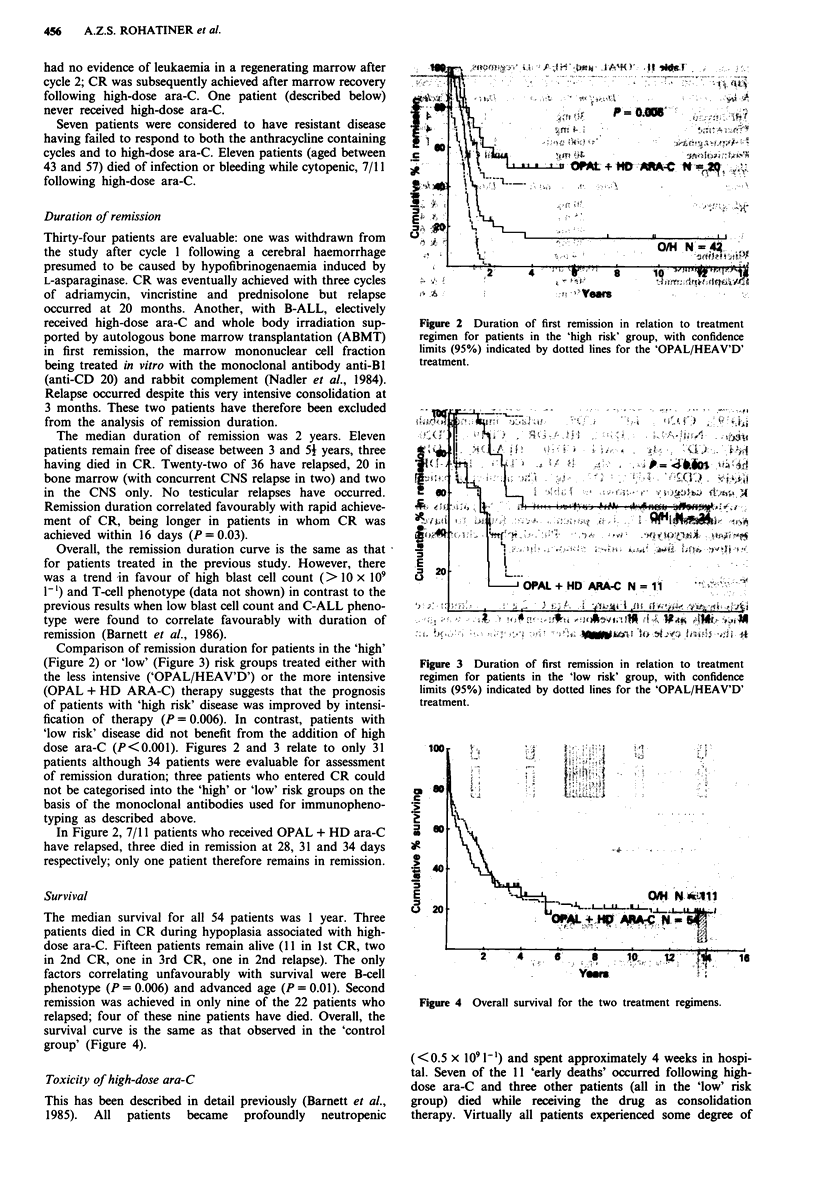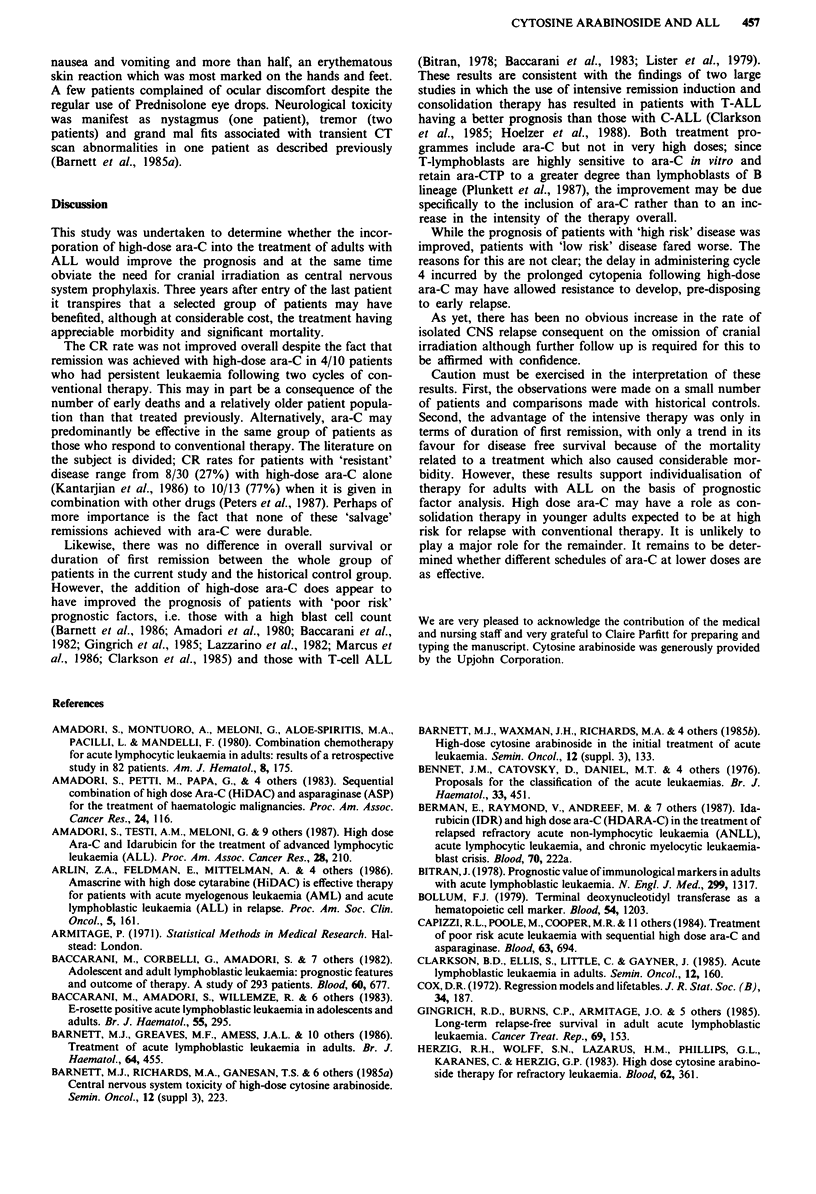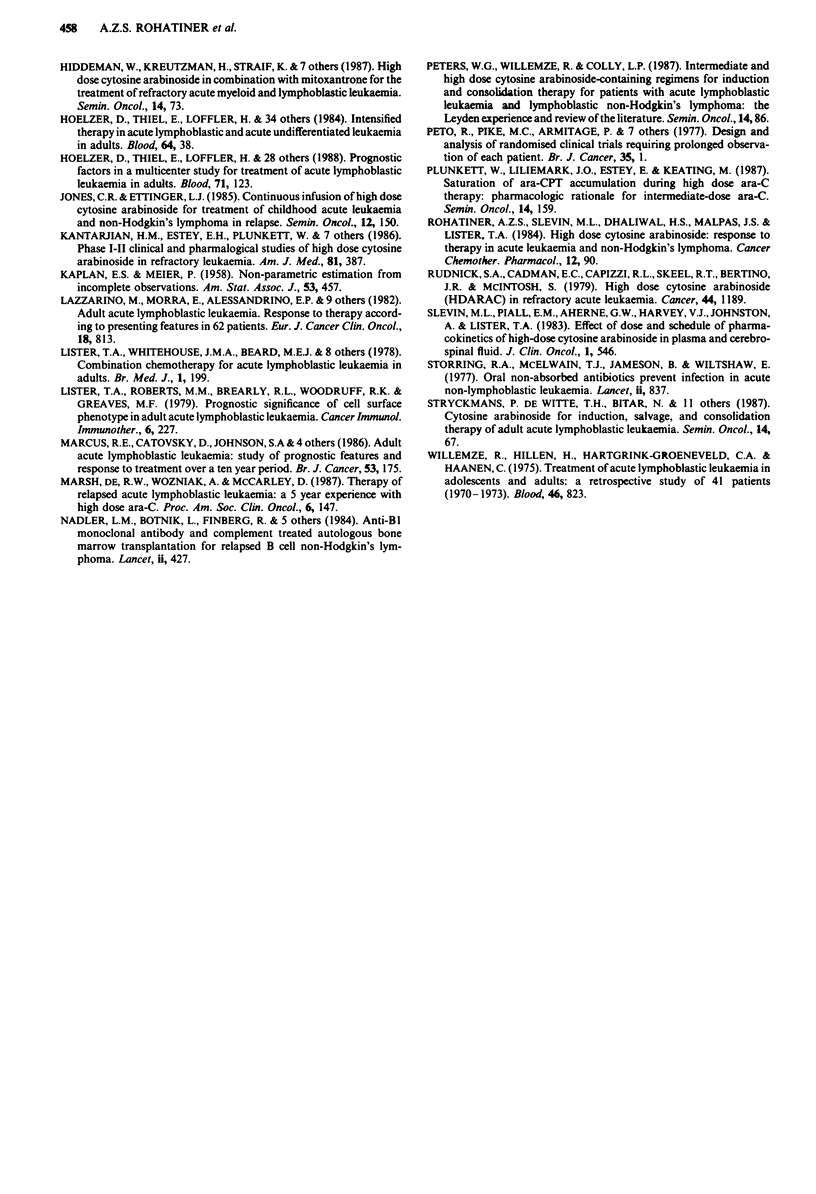# High dose cytosine arabinoside in the initial treatment of adults with acute lymphoblastic leukaemia.

**DOI:** 10.1038/bjc.1990.317

**Published:** 1990-09

**Authors:** A. Z. Rohatiner, R. Bassan, R. Battista, M. J. Barnett, W. Gregory, J. Lim, J. Amess, A. Oza, T. Barbui, M. Horton

**Affiliations:** ICRF Department of Medical Oncology, St Bartholowmew's Hospital, West Smithfield, London, UK.

## Abstract

In a study conducted at St Bartholomew's Hospital between 1972 and 1982, using moderately intensive therapy (OPAL/HEAV'D), a low blast count at presentation (less than 10 x 10(9) 1(-1)) and common ALL (C-ALL) phenotype correlated favourably with duration of remission. Fifty-four patients (age range 15-57, median 32) subsequently received a modification of the previous treatment programme which included high-dose ara-C 2 g m-2 b.d. for 6 days as cycle 3 (OPAL + HD ARA-C). CR was achieved in 36/54 (67%) patients, response correlating favourably with younger age (15-30 years vs 31-57 years, P = 0.02). Three patients died in CR. Overall, there was no difference in survival or remission duration between patients who received high dose ara-C and those in the control group. However, in contrast to the early results, there was a reversal in the relevance of the prognostic factors with a trend in favour of high blast count (greater than 10 x 10(9) 1(-1)) and T-cell phenotype in terms of remission duration. Moreover, comparison of duration of remission for the previously defined prognostic groups according to therapy suggests that the prognosis of patients with 'high risk' disease (T, B, null ALL or high blast count) is improved with more intensive therapy. In contrast, those with 'low risk' disease (C-ALL and low blast count) have a better prognosis with less intensive therapy. These observations confirm those of others and allow for individualization of therapy on the basis of pre-treatment variables.


Br. J. Cancer (1990), 62, 454-458 © Macmillan Press Ltd., 1990

### High dose cytosine arabinoside in the initial treatment of adults with acute lymphoblastic leukaemia

A.Z.S. Rohatiner[1], R. Bassan[2], R. Battista[3], M.J. Barnett[1], W. Gregory[1], J. Lim[1], J. Amess[4], A. Oza[1], T. Barbui[2], M. Horton[4], T. Chisesi[3] & T.A. Lister[1]

[1]*ICRF Department of Medical Oncology, St Bartholomew's Hospital, West Smithfield, London EC1A 7BE, UK;* [2]*Ospedale Riuniti, Bergamo, Italy;* [3]*Ospedale San Bortolo, Vincenza, Italy; and* [4]*Department of Haematology, St Bartholomew's Hospital, West Smithfield, London EC1A 7BE, UK.*

**Summary** In a study conducted at St Bartholomew's Hospital between 1972 and 1982, using moderately intensive therapy (OPAL/HEAV'D), a low blast count at presentation ($<10 \times 10^9 l^{-1}$) and common ALL (C-ALL) phenotype correlated favourably with duration of remission. Fifty-four patients (age range 15–57, median 32) subsequently received a modification of the previous treatment programme which included high-dose ara-C 2 g m$^{-2}$ b.d. for 6 days as cycle 3 (OPAL + HD ARA-C). CR was achieved in 36/54 (67%) patients, response correlating favourably with younger age (15–30 years *vs* 31–57 years, $P = 0.02$). Three patients died in CR. Overall, there was no difference in survival or remission duration between patients who received high dose ara-C and those in the control group. However, in contrast to the early results, there was a reversal in the relevance of the prognostic factors with a trend in favour of high blast count ($> 10 \times 10^9 l^{-1}$) and T-cell phenotype in terms of remission duration. Moreover, comparison of duration of remission for the previously defined prognostic groups according to therapy suggests that the prognosis of patients with 'high risk' disease (T, B, null ALL or high blast count) is improved with more intensive therapy. In contrast, those with 'low risk' disease (C-ALL and low blast count) have a better prognosis with less intensive therapy. These observations confirm those of others and allow for individualisation of therapy on the basis of pre-treatment variables.

Complete remission (CR) can be achieved in the majority of adults with acute lymphoblastic leukaemia (ALL) with standard combination chemotherapy yet only a minority are cured (Willemze *et al.*, 1975, Hoelzer *et al.*, 1984; Barnett *et al.*, 1986). Intensive consolidation therapy resulting in more effective elimination of 'minimal residual disease' might thus be expected to reduce the frequency of relapse.

Cytosine arabinoside (ara-C) in high doses, alone (Rudnick *et al.*, 1979; Herzig *et al.*, 1983; Rohatiner *et al.*, 1984; Kantarjian *et al.*, 1984; Marsh *et al.*, 1987) and in combination with other drugs (Amadori *et al.*, 1983, 1987; Capizzi *et al.*, 1984; Jones *et al.*, 1985; Arlin *et al.*, 1986; Stryckmans *et al.*, 1987; Hiddeman *et al.*, 1987; Peters *et al.*, 1987; Berman *et al.*, 1987) has been shown to induce remission in refractory and recurrent ALL although the remissions are short lived. Furthermore, it has been demonstrated that therapeutic levels of ara-C can be maintained in the cerebro-spinal fluid after systemic administration of the drug at high dosage (Slevin *et al.*, 1983). Thus 'high dose' ara-C might be exploited both as systemic anti-leukaemic therapy and as potential central nervous system (CNS) prophylaxis in patients with ALL.

Against this background, ara-C was incorporated into a treatment programme for newly diagnosed patients with ALL in the hope of prolonging remission duration and hence survival. The results of this approach are presented below and contrasted with the results of two earlier, less intensive treatment programmes.

### Materials and methods

### *Patients*

Between January 1983 and October 1986, 54 newly diagnosed patients were treated at three centres: St Bartholomew's Hospital (SBH), London (30 patients), Ospedale Riuniti, Bergamo and Ospedale San Bortolo, Vicenza (24 patients). Their clinical characteristics are shown in Table I. Patients with a blast count $< 10 \times 10^9 l^{-1}$ in whom the phenotype was unknown (OPAL + HD ara-C, five patients, OPAL/HEAV'D, 19 patients) either because an aspirate could not be obtained or, in the case of patients treated in 1972 and 1973 because phenotyping was not done, are not included in Table I.

Comparison is made with the outcome for 111 patients of similar age distribution treated at SBH between 1972 and 1982 with two treatment programmes, 'OPAL' and 'HEAV'D', as previously reported (Lister *et al.*, 1978; Barnett *et al.*, 1986) and patients who subsequently received 'OPAL' between November 1986 and November 1987. Details of these two regimens are shown in Table II.

### *Diagnosis*

The morphological diagnosis was based on May-Grunwald-Giemsa and cytochemical staining of bone marrow smears which showed infiltration by at least 30% lymphoblasts. All were classified according to the FAB system (Bennett *et al.*, 1976). Blast cells were isolated by standard density gradient centrifugation and immunophenotyping studies performed using a panel of monoclonal antibodies. A percentage bind-

**Table I** Clinical characteristics of patients in the 'high' and 'low' risk groups at presentation

| | *OPAL + H/D ARA-C* | | *OPAL/HEAV'D* | |
|---|---|---|---|---|
| | *'High'* | *'Low'* | *'High'* | *'Low'* |
| Age | | | | |
| Range | 15–57 | 15–57 | 15–58 | 16–52 |
| Mean | 32 | 34-69 | 30 | 29 |
| Median | 29 | 37 | 23 | 26 |
| Absolute blast count | | | | |
| Range | 0–355 | 0–9.1 | 0–435 | 0–9.5 |
| Median | 13.2 | 0.9 | 22 | 1.3 |
| Phenotype | | | | |
| C | 7 | 13 | 14 | 29 |
| Null | 13 | – | 23 | – |
| B | 5 | – | 6 | – |
| T | 9 | – | 15 | – |
| Other | 1 | – | 5 | – |
| Not done | 1 | – | – | |
| Total | 36 | 13 | 63 | 29 |

Correspondence: A.Z.S. Rohatiner.
Received 2 November 1989; and in revised form 28 February 1990.

**Table II** 'OPAL' and 'HEAV'D' regimens

| *'OPAL'* *Drug* | *Dose($m^{-2}$) (daily)* | *Days* | *Cycles* |
|---|---|---|---|
| Adriamycin | 30 mg | 1 | 4 |
| Vincristine | 1.4 mg | 1 | 4 |
| L-Asparaginase | 10,000 units | 1-14 | 1 |
| Prednisolone | 40 mg | until CR | |
| *'HEAV'D'* *Drug* | *Dose ($m^{-2}$) (daily)* | *Days* | *Cycles* |
| Adriamycin | 30 mg | 1 | 1 & 2 |
| | 25 mg | 1-3 | 3 & 4 |
| | 40 mg | 1 | 5 & 6 |
| | 30 mg | 2-3 | 5 & 6 |
| Vincristine | 1.4 mg | 1 | 1-6 |
| L-Asparaginase | 10,000 units | 1-14 | 1 |
| Cyclophosphamide | 500 mg | 1 | 3 & 4 |
| | 750 mg | 1 | 5 & 6 |
| Prednisolone | 40 mg | until CR | |

ing of greater than 20% was considered positive. Samples were also tested for terminal deoxynucleotidyl transferase (TdT) (Bollum, 1979). The results were interpreted according to the following classification. C-ALL: $CD10^+$, $HLA\text{-}DR^+$, $CD19^+$, $CD20^-$, $TdT^+$, $CD7^-$, surface immunoglobulin $(sIg)^-$. Null-ALL: $CD10^-$, $HLA\text{-}DR^+$, $CD19^-$, $CD20^-$, $TdT^+$, $CD7^-$, $sIg^-$. T-ALL: $CD10^-$, $HLA\text{-}DR^-$, $CD19^-$, $CD20^-$, $TdT^+$, $CD7^+$, $sIg^-$. B-ALL: $CD10^-$, $HLA\text{-}DR^+$, $CD19^-$, $CD20^+$, $TdT^-$, $CD7^-$, $sIg^+$. The number of patients in each category is shown in Table I.

Cytogenetic analysis was carried out in 27/54 patients and was successful in 17. Ten patients were found to have a normal karyotype, two were Philadelphia chromosome positive and five had other abnormalities.

### *Treatment*

Details are shown in Figure 1. Ara-C: $2\,g\,m^{-2}$, administered twice daily as a 3-h intravenous infusion for 6 days was given as the third cycle of treatment after the peripheral blood and bone marrow had recovered following cycle 2. In the majority of patients, the interval between cycles 2 and 3 was 2 weeks. Ara-C was administered irrespective of whether CR had been achieved. In 2/9 patients who were over the age of 50, the dose of ara-C was reduced to $1.5\,g\,m^{-2}$ b.d. Prednisolone eye drops were prescribed every 2 h for 10 days from the commencement of ara-C.

CNS prophylaxis (in addition to the systemic ara-C) comprised intrathecal methotrexate (MTX) 12.5 mg, given as soon as leukaemic blasts cells had cleared from the peripheral blood and with each cycle of adriamycin and vincristine thereafter. Intrathecal MTX or ara-C were subsequently administered every 2 months for 2 years. No cranial irradiation was given.

Maintenance therapy comprised 6-mercaptopurine daily and cyclophosphamide and MTX weekly to maintain the white cell count below $3 \times 10^9\,l^{-1}$, for a total of 3 years.

**Figure 1** OPAL + H/D ara-C regimen.

### *Supportive care*

Patients spent the first and third cycles of treatment in hospital on an open ward. The second, fourth and fifth cycles were generally given on an outpatient basis. Prophylactic oral non-absorbable antibiotics (Storring *et al.*, 1977) were prescribed from the onset of treatment. Platelet transfusions from single donors were given prophylactically to maintain the platelet count above $20 \times 10^9\,l^{-1}$ or if clinically indicated. Fever was assumed to be bacterial in origin and was treated with an aminoglycoside/cephalosporin combination in the first instance.

### *Definitions*

*Complete remission (CR)* CR required the patient to be in normal health, with a haemoglobin concentration greater than $10\,g\,dl^{-1}$, neutrophils greater than $1.0 \times 10^9\,l^{-1}$, and platelets greater than $100 \times 10^9\,l^{-1}$; the bone marrow to be normocellular, with representation of all cell lines in normal numbers and no leukaemic blast cells; and a CSF cytocentrifuge specimen to contain no blast cells.

*'Risk'* Patients were grouped on the basis of prognostic factors derived from the previous analysis (Barnett *et al.*, 1986). 'High risk': T, B, null-ALL or blast count $>10 \times 10^9\,l^{-1}$ at presentation. 'Low risk': C-ALL and blast count $<10 \times 10^9\,l^{-1}$ at presentation.

### *Statistical analysis*

Proportions of patients achieving CR in different prognostic groups were compared using the $\chi^2$ test with Yate's correction (Armitage, 1971). Duration of remission and overall survival were plotted using standard life table methods (Kaplan & Meier, 1958) and compared using the log rank method (Peto *et al.*, 1977). The significance of prognostic factors in determining the achievement of CR was evaluated by logistic regression analysis, whereas duration of CR and overall survival differences were determined using a stepwise linear regression method based on Cox's proportional hazards model (Cox, 1972).

### Results

### *Response to therapy*

CR was achieved in 36/54 (67%) patients overall, response correlating favourably with younger age (15-30 years *vs* 31-57 years, $P=0.02$) and unfavourably with B-cell phenotype ($P<0.04$). The absolute blast count at presentation did not correlate with response.

CR was achieved before commencing high-dose ara-C in 25/54 patients and subsequently in ten patients. Five of the latter had overt residual leukaemia before receiving high dose ara-C; in four of these five, CR was documented after marrow recovery following high dose ara-C. The patient who did not enter CR with high-dose ara-C eventually did so with a further cycle of adriamycin and vincristine. However, in all five patients the remissions were short. A further five patients had no evidence of leukaemia in a regenerating marrow after cycle 2; CR was subsequently achieved after marrow recovery following high-dose ara-C. One patient (described below) never received high-dose ara-C.

Seven patients were considered to have resistant disease having failed to respond to both the anthracycline containing cycles and to high-dose ara-C. Eleven patients (aged between 43 and 57) died of infection or bleeding while cytopenic, 7/11 following high-dose ara-C.

*Duration of remission*

Thirty-four patients are evaluable: one was withdrawn from the study after cycle 1 following a cerebral haemorrhage presumed to be caused by hypofibrinogenaemia induced by L-asparaginase. CR was eventually achieved with three cycles of adriamycin, vincristine and prednisolone but relapse occurred at 20 months. Another, with B-ALL, electively received high-dose ara-C and whole body irradiation supported by autologous bone marrow transplantation (ABMT) in first remission, the marrow mononuclear cell fraction being treated *in vitro* with the monoclonal antibody anti-B1 (anti-CD 20) and rabbit complement (Nadler *et al.*, 1984). Relapse occurred despite this very intensive consolidation at 3 months. These two patients have therefore been excluded from the analysis of remission duration.

The median duration of remission was 2 years. Eleven patients remain free of disease between 3 and 5½ years, three having died in CR. Twenty-two of 36 have relapsed, 20 in bone marrow (with concurrent CNS relapse in two) and two in the CNS only. No testicular relapses have occurred. Remission duration correlated favourably with rapid achievement of CR, being longer in patients in whom CR was achieved within 16 days ($P = 0.03$).

Overall, the remission duration curve is the same as that for patients treated in the previous study. However, there was a trend in favour of high blast cell count ($> 10 \times 10^9$ $l^{-1}$) and T-cell phenotype (data not shown) in contrast to the previous results when low blast cell count and C-ALL phenotype were found to correlate favourably with duration of remission (Barnett *et al.*, 1986).

Comparison of remission duration for patients in the 'high' (Figure 2) or 'low' (Figure 3) risk groups treated either with the less intensive ('OPAL/HEAV'D') or the more intensive (OPAL + HD ARA-C) therapy suggests that the prognosis of patients with 'high risk' disease was improved by intensification of therapy ($P = 0.006$). In contrast, patients with 'low risk' disease did not benefit from the addition of high dose ara-C ($P < 0.001$). Figures 2 and 3 relate to only 31 patients although 34 patients were evaluable for assessment of remission duration; three patients who entered CR could not be categorised into the 'high' or 'low' risk groups on the basis of the monoclonal antibodies used for immunophenotyping as described above.

In Figure 2, 7/11 patients who received OPAL + HD ara-C have relapsed, three died in remission at 28, 31 and 34 days respectively; only one patient therefore remains in remission.

**Figure 2** Duration of first remission in relation to treatment regimen for patients in the 'high risk' group, with confidence limits (95%) indicated by dotted lines for the 'OPAL/HEAV'D' treatment.

**Figure 3** Duration of first remission in relation to treatment regimen for patients in the 'low risk' group, with confidence limits (95%) indicated by dotted lines for the 'OPAL/HEAV'D' treatment.

**Figure 4** Overall survival for the two treatment regimens.

*Survival*

The median survival for all 54 patients was 1 year. Three patients died in CR during hypoplasia associated with high-dose ara-C. Fifteen patients remain alive (11 in 1st CR, two in 2nd CR, one in 3rd CR, one in 2nd relapse). The only factors correlating unfavourably with survival were B-cell phenotype ($P = 0.006$) and advanced age ($P = 0.01$). Second remission was achieved in only nine of the 22 patients who relapsed; four of these nine patients have died. Overall, the survival curve is the same as that observed in the 'control group' (Figure 4).

*Toxicity of high-dose ara-C*

This has been described in detail previously (Barnett *et al.*, 1985). All patients became profoundly neutropenic ($< 0.5 \times 10^9\ l^{-1}$) and spent approximately 4 weeks in hospital. Seven of the 11 'early deaths' occurred following high-dose ara-C and three other patients (all in the 'low' risk group) died while receiving the drug as consolidation therapy. Virtually all patients experienced some degree of nausea and vomiting and more than half, an erythematous skin reaction which was most marked on the hands and feet. A few patients complained of ocular discomfort despite the regular use of Prednisolone eye drops. Neurological toxicity was manifest as nystagmus (one patient), tremor (two patients) and grand mal fits associated with transient CT scan abnormalities in one patient as described previously (Barnett *et al*., 1985*a*).

### Discussion

This study was undertaken to determine whether the incorporation of high-dose ara-C into the treatment of adults with ALL would improve the prognosis and at the same time obviate the need for cranial irradiation as central nervous system prophylaxis. Three years after entry of the last patient it transpires that a selected group of patients may have benefited, although at considerable cost, the treatment having appreciable morbidity and significant mortality.

The CR rate was not improved overall despite the fact that remission was achieved with high-dose ara-C in 4/10 patients who had persistent leukaemia following two cycles of conventional therapy. This may in part be a consequence of the number of early deaths and a relatively older patient population than that treated previously. Alternatively, ara-C may predominantly be effective in the same group of patients as those who respond to conventional therapy. The literature on the subject is divided; CR rates for patients with 'resistant' disease range from 8/30 (27%) with high-dose ara-C alone (Kantarjian *et al*., 1986) to 10/13 (77%) when it is given in combination with other drugs (Peters *et al*., 1987). Perhaps of more importance is the fact that none of these 'salvage' remissions achieved with ara-C were durable.

Likewise, there was no difference in overall survival or duration of first remission between the whole group of patients in the current study and the historical control group. However, the addition of high-dose ara-C does appear to have improved the prognosis of patients with 'poor risk' prognostic factors, i.e. those with a high blast cell count (Barnett *et al*., 1986; Amadori *et al*., 1980; Baccarani *et al*., 1982; Gingrich *et al*., 1985; Lazzarino *et al*., 1982; Marcus *et al*., 1986; Clarkson *et al*., 1985) and those with T-cell ALL (Bitran, 1978; Baccarani *et al*., 1983; Lister *et al*., 1979). These results are consistent with the findings of two large studies in which the use of intensive remission induction and consolidation therapy has resulted in patients with T-ALL having a better prognosis than those with C-ALL (Clarkson *et al*., 1985; Hoelzer *et al*., 1988). Both treatment programmes include ara-C but not in very high doses; since T-lymphoblasts are highly sensitive to ara-C *in vitro* and retain ara-CTP to a greater degree than lymphoblasts of B lineage (Plunkett *et al*., 1987), the improvement may be due specifically to the inclusion of ara-C rather than to an increase in the intensity of the therapy overall.

While the prognosis of patients with 'high risk' disease was improved, patients with 'low risk' disease fared worse. The reasons for this are not clear; the delay in administering cycle 4 incurred by the prolonged cytopenia following high-dose ara-C may have allowed resistance to develop, pre-disposing to early relapse.

As yet, there has been no obvious increase in the rate of isolated CNS relapse consequent on the omission of cranial irradiation although further follow up is required for this to be affirmed with confidence.

Caution must be exercised in the interpretation of these results. First, the observations were made on a small number of patients and comparisons made with historical controls. Second, the advantage of the intensive therapy was only in terms of duration of first remission, with only a trend in its favour for disease free survival because of the mortality related to a treatment which also caused considerable morbidity. However, these results support individualisation of therapy for adults with ALL on the basis of prognostic factor analysis. High dose ara-C may have a role as consolidation therapy in younger adults expected to be at high risk for relapse with conventional therapy. It is unlikely to play a major role for the remainder. It remains to be determined whether different schedules of ara-C at lower doses are as effective.

We are very pleased to acknowledge the contribution of the medical and nursing staff and very grateful to Claire Parfitt for preparing and typing the manuscript. Cytosine arabinoside was generously provided by the Upjohn Corporation.

### References

AMADORI, S., MONTUORO, A., MELONI, G., ALOE-SPIRITIS, M.A., PACILLI, L. & MANDELLI, F. (1980). Combination chemotherapy for acute lymphocytic leukaemia in adults: results of a retrospective study in 82 patients. *Am. J. Hematol*., **8**, 175.

AMADORI, S., PETTI, M., PAPA, G., & 4 others (1983). Sequential combination of high dose Ara-C (HiDAC) and asparaginase (ASP) for the treatment of haematologic malignancies. *Proc. Am. Assoc. Cancer Res*., **24**, 116.

AMADORI, S., TESTI, A.M., MELONI, G. & 9 others (1987). High dose Ara-C and Idarubicin for the treatment of advanced lymphocytic leukaemia (ALL). *Proc. Am. Assoc. Cancer Res*., **28**, 210.

ARLIN, Z.A., FELDMAN, E., MITTELMAN, A. & 4 others (1986). Amascrine with high dose cytarabine (HiDAC) is effective therapy for patients with acute myelogenous leukaemia (AML) and acute lymphoblastic leukaemia (ALL) in relapse. *Proc. Am. Soc. Clin. Oncol*., **5**, 161.

ARMITAGE, P. (1971). *Statistical Methods in Medical Research*. Halstead: London.

BACCARANI, M., CORBELLI, G., AMADORI, S. & 7 others (1982). Adolescent and adult lymphoblastic leukaemia: prognostic features and outcome of therapy. A study of 293 patients. *Blood*, **60**, 677.

BACCARANI, M., AMADORI, S., WILLEMZE, R. & 6 others (1983). E-rosette positive acute lymphoblastic leukaemia in adolescents and adults. *Br. J. Haematol*., **55**, 295.

BARNETT, M.J., GREAVES, M.F., AMESS, J.A.L. & 10 others (1986). Treatment of acute lymphoblastic leukaemia in adults. *Br. J. Haematol*., **64**, 455.

BARNETT, M.J., RICHARDS, M.A., GANESAN, T.S. & 6 others (1985*a*) Central nervous system toxicity of high-dose cytosine arabinoside. *Semin. Oncol*., **12** (suppl 3), 223.

BARNETT, M.J., WAXMAN, J.H., RICHARDS, M.A. & 4 others (1985*b*). High-dose cytosine arabinoside in the initial treatment of acute leukaemia. *Semin. Oncol*., **12** (suppl. 3), 133.

BENNET, J.M., CATOVSKY, D., DANIEL, M.T. & 4 others (1976). Proposals for the classification of the acute leukaemias. *Br. J. Haematol*., **33**, 451.

BERMAN, E., RAYMOND, V., ANDREEF, M. & 7 others (1987). Idarubicin (IDR) and high dose ara-C (HDARA-C) in the treatment of relapsed refractory acute non-lymphocytic leukaemia (ANLL), acute lymphocytic leukaemia, and chronic myelocytic leukaemia-blast crisis. *Blood*, **70**, 222a.

BITRAN, J. (1978). Prognostic value of immunological markers in adults with acute lymphoblastic leukaemia. *N. Engl. J. Med*., **299**, 1317.

BOLLUM, F.J. (1979). Terminal deoxynucleotidyl transferase as a hematopoietic cell marker. *Blood*, **54**, 1203.

CAPIZZI, R.L., POOLE, M., COOPER, M.R. & 11 others (1984). Treatment of poor risk acute leukaemia with sequential high dose ara-C and asparaginase. *Blood*, **63**, 694.

CLARKSON, B.D., ELLIS, S., LITTLE, C. & GAYNER, J. (1985). Acute lymphoblastic leukaemia in adults. *Semin. Oncol*., **12**, 160.

COX, D.R. (1972). Regression models and lifetables. *J. R. Stat. Soc. (B)*, **34**, 187.

GINGRICH, R.D., BURNS, C.P., ARMITAGE, J.O. & 5 others (1985). Long-term relapse-free survival in adult acute lymphoblastic leukaemia. *Cancer Treat. Rep*., **69**, 153.

HERZIG, R.H., WOLFF, S.N., LAZARUS, H.M., PHILLIPS, G.L., KARANES, C. & HERZIG, G.P. (1983). High dose cytosine arabinoside therapy for refractory leukaemia. *Blood*, **62**, 361.

HIDDEMAN, W., KREUTZMAN, H., STRAIF, K. & 7 others (1987). High dose cytosine arabinoside in combination with mitoxantrone for the treatment of refractory acute myeloid and lymphoblastic leukaemia. *Semin. Oncol.*, **14**, 73.

HOELZER, D., THIEL, E., LOFFLER, H. & 34 others (1984). Intensified therapy in acute lymphoblastic and acute undifferentiated leukaemia in adults. *Blood*, **64**, 38.

HOELZER, D., THIEL, E., LOFFLER, H. & 28 others (1988). Prognostic factors in a multicenter study for treatment of acute lymphoblastic leukaemia in adults. *Blood*, **71**, 123.

JONES, C.R. & ETTINGER, L.J. (1985). Continuous infusion of high dose cytosine arabinoside for treatment of childhood acute leukaemia and non-Hodgkin's lymphoma in relapse. *Semin. Oncol.*, **12**, 150.

KANTARJIAN, H.M., ESTEY, E.H., PLUNKETT, W. & 7 others (1986). Phase I-II clinical and pharmalogical studies of high dose cytosine arabinoside in refractory leukaemia. *Am. J. Med.*, **81**, 387.

KAPLAN, E.S. & MEIER, P. (1958). Non-parametric estimation from incomplete observations. *Am. Stat. Assoc. J.*, **53**, 457.

LAZZARINO, M., MORRA, E., ALESSANDRINO, E.P. & 9 others (1982). Adult acute lymphoblastic leukaemia. Response to therapy according to presenting features in 62 patients. *Eur. J. Cancer Clin. Oncol.*, **18**, 813.

LISTER, T.A., WHITEHOUSE, J.M.A., BEARD, M.E.J. & 8 others (1978). Combination chemotherapy for acute lymphoblastic leukaemia in adults. *Br. Med. J.*, **1**, 199.

LISTER, T.A., ROBERTS, M.M., BREARLY, R.L., WOODRUFF, R.K. & GREAVES, M.F. (1979). Prognostic significance of cell surface phenotype in adult acute lymphoblastic leukaemia. *Cancer Immunol. Immunother.*, **6**, 227.

MARCUS, R.E., CATOVSKY, D., JOHNSON, S.A & 4 others (1986). Adult acute lymphoblastic leukaemia: study of prognostic features and response to treatment over a ten year period. *Br. J. Cancer*, **53**, 175.

MARSH, DE, R.W., WOZNIAK, A. & MCCARLEY, D. (1987). Therapy of relapsed acute lymphoblastic leukaemia: a 5 year experience with high dose ara-C. *Proc. Am. Soc. Clin. Oncol.*, **6**, 147.

NADLER, L.M., BOTNIK, L., FINBERG, R. & 5 others (1984). Anti-B1 monoclonal antibody and complement treated autologous bone marrow transplantation for relapsed B cell non-Hodgkin's lymphoma. *Lancet*, **ii**, 427.

PETERS, W.G., WILLEMZE, R. & COLLY, L.P. (1987). Intermediate and high dose cytosine arabinoside-containing regimens for induction and consolidation therapy for patients with acute lymphoblastic leukaemia and lymphoblastic non-Hodgkin's lymphoma: the Leyden experience and review of the literature. *Semin. Oncol.*, **14**, 86.

PETO, R., PIKE, M.C., ARMITAGE, P. & 7 others (1977). Design and analysis of randomised clinical trials requiring prolonged observation of each patient. *Br. J. Cancer*, **35**, 1.

PLUNKETT, W., LILIEMARK, J.O., ESTEY, E. & KEATING, M. (1987). Saturation of ara-CPT accumulation during high dose ara-C therapy: pharmacologic rationale for intermediate-dose ara-C. *Semin. Oncol.*, **14**, 159.

ROHATINER, A.Z.S., SLEVIN, M.L., DHALIWAL, H.S., MALPAS, J.S. & LISTER, T.A. (1984). High dose cytosine arabinoside: response to therapy in acute leukaemia and non-Hodgkin's lymphoma. *Cancer Chemother. Pharmacol.*, **12**, 90.

RUDNICK, S.A., CADMAN, E.C., CAPIZZI, R.L., SKEEL, R.T., BERTINO, J.R. & MCINTOSH, S. (1979). High dose cytosine arabinoside (HDARAC) in refractory acute leukaemia. *Cancer*, **44**, 1189.

SLEVIN, M.L., PIALL, E.M., AHERNE, G.W., HARVEY, V.J., JOHNSTON, A. & LISTER, T.A. (1983). Effect of dose and schedule of pharmacokinetics of high-dose cytosine arabinoside in plasma and cerebrospinal fluid. *J. Clin. Oncol.*, **1**, 546.

STORRING, R.A., MCELWAIN, T.J., JAMESON, B. & WILTSHAW, E. (1977). Oral non-absorbed antibiotics prevent infection in acute non-lymphoblastic leukaemia. *Lancet*, **ii**, 837.

STRYCKMANS, P. DE WITTE, T.H., BITAR, N. & 11 others (1987). Cytosine arabinoside for induction, salvage, and consolidation therapy of adult acute lymphoblastic leukaemia. *Semin. Oncol.*, **14**, 67.

WILLEMZE, R., HILLEN, H., HARTGRINK-GROENEVELD, C.A. & HAANEN, C. (1975). Treatment of acute lymphoblastic leukaemia in adolescents and adults: a retrospective study of 41 patients (1970–1973). *Blood*, **46**, 823.